# Quantitative Proteome Profiling of a *S*-Nitrosoglutathione Reductase (GSNOR) Null Mutant Reveals a New Class of Enzymes Involved in Nitric Oxide Homeostasis in Plants

**DOI:** 10.3389/fpls.2021.787435

**Published:** 2021-12-07

**Authors:** Patrick Treffon, Jacopo Rossi, Giuseppe Gabellini, Paolo Trost, Mirko Zaffagnini, Elizabeth Vierling

**Affiliations:** ^1^Department of Biochemistry and Molecular Biology, University of Massachusetts Amherst, Amherst, MA, United States; ^2^Department of Pharmacy and Biotechnologies, University of Bologna, Bologna, Italy

**Keywords:** *Arabidopsis*, *hot5-2*, nitric oxide homeostasis, aldo-keto reductases, *S*-nitrosoglutathione reductase, *S*-nitrosoglutathione, *S*-nitroso-CoA, protein S-nitrosylation/S-nitrosation

## Abstract

Nitric oxide (NO) is a short-lived radical gas that acts as a signaling molecule in all higher organisms, and that is involved in multiple plant processes, including germination, root growth, and fertility. Regulation of NO-levels is predominantly achieved by reaction of oxidation products of NO with glutathione to form *S*-nitrosoglutathione (GSNO), the principal bioactive form of NO. The enzyme *S*-nitrosoglutathione reductase (GSNOR) is a major route of NADH-dependent GSNO catabolism and is critical to NO homeostasis. Here, we performed a proteomic analysis examining changes in the total leaf proteome of an *Arabidopsis thaliana* GSNOR null mutant (*hot5-2/gsnor1-3*). Significant increases or decreases in proteins associated with chlorophyll metabolism and with redox and stress metabolism provide insight into phenotypes observed in *hot5-2/gsnor1-3* plants. Importantly, we identified a significant increase in proteins that belong to the aldo-keto reductase (AKR) protein superfamily, AKR4C8 and 9. Because specific AKRs have been linked to NO metabolism in mammals, we expressed and purified *A. thaliana* AKR4C8 and 9 and close homologs AKR4C10 and 11 and determined that they have NADPH-dependent activity in GSNO and *S*-nitroso-coenzyme A (SNO-CoA) reduction. Further, we found an increase of NADPH-dependent GSNO reduction activity in *hot5-2/gsnor1-3* mutant plants. These data uncover a new, NADPH-dependent component of NO metabolism that may be integrated with NADH-dependent GSNOR activity to control NO homeostasis in plants.

## Introduction

The ubiquitous signaling molecule nitric oxide (NO) is a free radical gas and constitutes the prominent reactive nitrogen species (RNS) in cells. In plants, RNS are involved in regulating physiological and developmental processes including stomatal movement (Bellin et al., 2013; Fu et al., 2016), fertility (Duan et al., 2020; Wang et al., 2021), germination (Nonogaki, 2017), and plant–microbe interactions (Matamoros et al., 2019). In addition, certain biotic and abiotic stresses induce NO production (Delledonne et al., 1998; Kneeshaw et al., 2014), linking it to plant hormone homeostasis including salicylic and jasmonic acid signaling (Rai et al., 2019; Zhang and Li, 2019), and ethylene- and auxin-dependent metabolism (Melo et al., 2016; Ni et al., 2017). The major signaling and regulatory effect of NO and other RNS is through reversible posttranslational modifications (PTMs). S-nitrosation (also referred to as nitrosylation), the addition of a NO group to reactive thiols of Cys residues of proteins, is reported to be the most important NO-related PTM (Astier et al., 2012; Guerra et al., 2016; Zaffagnini et al., 2016; Stomberski et al., 2019b). In addition, Tyr nitration and nitrosylation of metal-containing proteins are other PTMs associated with nitro-oxidative stresses (see Gupta et al., 2019 for NO PTM terminology). These NO-mediated PTMs can modulate protein activity, alter structural stability, and result in changes of protein subcellular localization or interaction with other proteins (Kovacs and Lindermayr, 2013; Guerra et al., 2016; Zhan et al., 2018; Feng et al., 2019; Zaffagnini et al., 2019).

The non-polar NO molecule can diffuse across biological membranes, but due to its short half-life (<6 s), its mode-of-action is local and restricted to areas of its production (Stamler et al., 1992; Thomas et al., 2001). However, oxidation products of NO (e.g., dinitrogen trioxide, N_2_O_3_) can spontaneously react with low molecular weight thiols like reduced glutathione (GSH) and coenzyme A (CoA), leading to the formation of the stable NO-carrying molecules nitrosoglutathione (GSNO) and nitroso-CoA (SNO-CoA), respectively. GSNO and SNO-CoA can be transported to distal regions and modify specific protein Cys targets (Anand et al., 2014; Jahnova et al., 2019; Stomberski et al., 2019b). Cytosolic/nuclear *S*-nitrosoglutathione reductase (GSNOR), a class III alcohol dehydrogenase, is a NADH-dependent enzyme that is involved in the regulation of GSNO levels (Liu et al., 2001; Feechan et al., 2005; Xu et al., 2013; Guerra et al., 2016). GSNOR decomposes GSNO to an *N*-hydroxysulphenamide intermediate (GSNHOH) that, under physiological conditions, is susceptible to nucleophilic attack by GSH, resulting in the formation of hydroxylamine (NH_2_OH) and oxidized glutathione (GSSG) (reviewed in Jahnova et al., 2019). Owing to the prominent role of GSNO as a *S*-nitrosating agent, GSNOR indirectly regulates the content of protein nitrosothiols (protein-SNOs) by irreversibly degrading GSNO and thereby fine-tuning the total amount of bioactive RNS *in planta* (Lindermayr, 2017). Interestingly, GSNO and other nitroso compounds (e.g., nitrosocysteine, Cys-NO) were found to induce S-nitrosation of plant GSNOR at solvent accessible Cys residues *in vitro* as well as *in vivo*, lowering its activity which could allow further NO signal propagation by reducing the breakdown of GSNO (Frungillo et al., 2014; Guerra et al., 2016; Chen et al., 2020). More recently, *Arabidopsis thaliana* catalase 3 (CAT3; AT1G20620) was demonstrated to induce trans-nitrosation of GSNOR at Cys10, impacting its stability and further indicating the importance of NO-dependent signaling in plants under normal and stress conditions (Chen et al., 2020). *A. thaliana* encodes a single-copy *GSNOR* gene (AT5G43940), and loss of the protein leads to elevated NO, nitrate, nitrite, and *S*-nitrosothiols (R-SNOs) and a proteome-wide increase in protein S-nitrosation (Feechan et al., 2005; Lee et al., 2008; Hu et al., 2015). In addition, *A. thaliana* GSNOR T-DNA null insertion alleles [*hot5-2* in Col-0 (also known as *atgsnor1-3*); *hot5-4* in the WS accession; hereafter referred to as *hot5-2*] exhibit multiple plant growth defects, including shorter and multi-branching inflorescences, reduced lateral roots, compromised pathogen response and a dramatic reduction in fertility (Feechan et al., 2005; Lee et al., 2008; Kwon et al., 2012; Wang et al., 2021). These phenotypes highlight that tightly controlled NO homeostasis is crucial for proper plant development.

Recently, Stamler and co-workers reported that a specific human aldo-keto reductase (AKR1A1; UniProt P14550), initially described to metabolize SNO-CoA (Zhou et al., 2019), is also involved in GSNO catabolism analogous to GSNOR (Stomberski et al., 2019b). Aldo-keto reductases (AKRs) are 34–37 kDa monomeric and NADPH-dependent oxidoreductases that share a common (α/β)_8_-barrel structural motif, a conserved cofactor binding domain and conserved catalytic tetrad. They act to decompose a broad range of reactive carbonyl substrates (Ford and Ellis, 2001; Hyndman et al., 2003; Sengupta et al., 2015). AKRs are widely distributed and typically reduce reactive ketones and aldehydes to the corresponding alcohols or perform the reverse oxidation reaction. The primary role of these enzymes may be to detoxify toxic compounds that arise during stress, since their expression is induced by different biotic and abiotic stresses (Vemanna et al., 2017; Niranjan et al., 2021). Notably, while GSNOR uses NADH as the reductant, AKR activity is strictly dependent on NADPH as a source of reducing equivalents (Fujii et al., 2021). Thus, AKR-dependent enzymatic reduction of GSNO, in addition to GSNOR activity, may participate in NO homeostasis to control NO-related biological functions under physiological and pathological conditions (Stomberski et al., 2019b; Zhou et al., 2019). However, the potential role of AKRs in NO/GSNO homeostasis in plants has not been explored.

Using quantitative proteome profiling, we report that leaves of *A. thaliana* GSNOR-null *hot5-2* mutant plants, which lack the central enzyme known to be involved in NO homeostasis, show an upregulation of specific AKR proteins. *In vitro* activity assays with the purified proteins demonstrate that the proteins metabolize GSNO and SNO-CoA in an NADPH-dependent manner. Further, NADPH-dependent GSNO degradation activity is increased in total protein extracts from leaves of *hot5-2* mutant plants. These findings strongly suggest that plants have an enzymatic system in addition to GSNOR that can modulate NO/GSNO levels.

## Materials and Methods

### Plant Material and Growth Conditions

*Arabidopsis thaliana* WT Col-0 and *hot5-2* (GABI_315D11) plants were used in this study and grown at 40–100 μmol m^–2^ s^–1^ at a 16 h light/8 h dark schedule and 22/18°C.

### Quantitative Proteomics

Leaves from 4- to 6-week-old soil grown WT Col-0 and *hot5-2* plants were used in the quantitative proteomics experiment. One biological replicate consists of nine plants and five biological replicates per genotype were used. Frozen leave plant material (200 mg) was ground in extraction buffer [25 mM HEPES-NaOH pH 7.7, 1 mM EDTA, 2.5% (w/v) SDS, protease inhibitor cocktail (Pierce A32955, Thermo Fisher Scientific, United States)] and subjected to trichloroacetic acid (TCA)/acetone precipitation. For that, 20% TCA (final concentration) were added to the protein extract and incubated for 45 min at −20°C. Proteins were sedimented by centrifugation at 16,000 × *g* for 10 min at 4°C and following three washes with ice-cold 70% acetone, proteins were resuspended in extraction buffer [25 mM HEPES-NaOH pH 7.7, 1 mM EDTA, 2.5% (w/v) SDS, protease inhibitor cocktail (Pierce A32955, Thermo Fisher Scientific, United States)]. Protein concentration was determined using a BCA assay (Pierce 23225, Thermo Fisher Scientific, United States). Fifty micrograms of total protein per sample was run for 15 min on an 4–20% SDS-PAGE system to separate proteins from lower molecular weight contaminants, and the entire protein region of the gel excised and subjected to in-gel trypsin digestion after reduction with 50 mM dithiothreitol (DTT, Sigma-Aldrich, United States) and subsequent alkylation with 100 mM iodoacetamide (IAM, Sigma-Aldrich, United States). Peptides eluted from the gel were lyophilized and resuspended in 20 μL of 0.1% (v/v) formic acid (FA). A 3 μL injection was loaded by a Thermo Easy nLC 1000 UPLC onto a 2 cm trapping column and desalted with 8 μL mobile phase A (0.1% FA in water). Peptides were then eluted at 300 nL min^–1^ onto a 75 μm i.d. × 50 cm RSLC column (Thermo Fisher Scientific, United States) using a linear gradient of 5–35% mobile phase B (0.1% FA in acetonitrile) over 150 min. Ions were introduced by positive electrospray ionization (ESI) using a stainless-steel capillary at 2.1 kV into a Thermo Orbitrap Fusion tribrid mass spectrometer. Mass spectra were acquired over m/z 300–1750 at 120,000 resolution (m/z 200) with an automatic gain target (AGC) of 1e6, a cycle time of 1 s, and data-dependent acquisition at top speed selecting the most abundant precursor ions for tandem mass spectrometry by higher-energy C-trap dissociation (HCD) fragmentation using an isolation width of 1.6 Da, maximum fill time 110 ms and AGC target 1e5. Peptides were fragmented with a normalized collision energy 27, and fragment ion spectra acquired at turbo speed in the linear ion trap. Dynamic exclusion was applied with an exclusion of 15 s after observing the same ion twice within 15 s.

### MS Data Analysis

Mass spectra were searched against the Uniprot *A. thaliana* databases (UP000006548_3702 and UP000006548_3702_additional, downloaded 07/2019) using MaxQuant version 1.6.7.0 with a 1% false discovery rate (FDR) at the peptide and protein level, peptides with a minimum length of seven amino acids with iodoacetamide-dependent cysteine carbamidomethylation, N-terminal acetylation, and methionine oxidation as fixed modifications. Enzyme specificity was set as C-terminal to arginine and lysine using trypsin as protease and a maximum of two missed cleavages were allowed in the database search. The maximum mass tolerance for precursor and fragment ions was 4.5 and 20 ppm, respectively, with second peptides and match between runs enabled. Label-free quantification (LFQ) was performed with the MaxLFQ algorithm (Cox et al., 2014) using a minimum ratio count of 2.

Identified protein groups generated by the MaxQuant program were uploaded to the Perseus program version 1.6.10.43 (Tyanova et al., 2016). Site only, reverse, and contaminant peptides were removed from the dataset and missing values were imputed using a normal distribution. Invalid values were then excluded, and empty columns were removed. The volcano plot function was used to identify proteins that were significantly changed using a *T*-test with a permutation-based FDR of 0.05 and an S0 of 0.1. For hierarchical clustering, LFQ intensities were first *z*-scored and significantly different proteins (two sample test, permutation-based FDR of 5%, 250 rounds of randomization) clustered using Euclidean as a distance measure for column and row clustering.

### Cloning, Protein Expression, and Purification of Recombinant Proteins

*Arabidopsis thaliana* AKR4C8 (AT2G37760.2), AKR4C9 (AT2G37770.2), AKR4C10 (AT2G37790.1), and AKR4C11 (AT3G53880.1) were cloned into pET23b-HIS_6_-SUMO vector (Liu et al., 2019) by Gibson Assembly using the primers listed in Supplementary Table 1. Expression and purification were performed as previously, with slight modifications (Dreyer et al., 2021). Ulp1 cleavage of the N-terminal HIS6-SUMO tag after immobilized metal affinity chromatography (IMAC) was performed during overnight dialysis against phosphate buffered saline (PBS) supplemented with 2 mM Tris(2-carboxyethyl) phosphine (TCEP, Sigma-Aldrich, United States). Following dialysis, samples were reapplied to Ni-NTA material (Thermo Fisher Scientific, United States) equilibrated in Tris–HCl pH 7.9 and the flow through, containing the non-tagged AKR4C proteins, was collected and concentrated by centrifugation if necessary. Protein molecular mass and purity were analyzed by SDS-PAGE after desalting with PD-10 columns (GE Healthcare, United States) equilibrated with 30 mM Tris–HCl pH 7.9. The concentration was determined spectrophotometrically using a molar extinction coefficient at 280 nm (ε_280_) of 50,420 M^–1^ cm^–1^ for AKR4C8, 61,420 M^–1^ cm^–1^ for AKR4C9, 59,930 M^–1^ cm^–1^ for AtAKR4C10, and 58,440 M^–1^ cm^–1^ for AtAKR4C11. The resulting homogeneous protein solutions were stored at −20°C.

### Enzyme Assays

*S*-nitrosoglutathione and CoA-SNO were prepared freshly as described previously (Stomberski et al., 2019b; Tagliani et al., 2021). Enzyme assays were performed in 100 mM Tris–HCl (pH 7.9) containing 0.2 mM NAD(P)H, 0.4 mM GSNO or SNO-CoA, and variable amounts of AKR4C proteins (50–200 nM) or *A. thaliana* leaf protein extracts (50–200 μg). Leaf protein extracts were obtained by resuspending 200 mg of plant material in extraction buffer [ratio 1:4 (w/v), 50 mM Tris–HCl (pH 7.9), 0.2% (v/v) Triton X-100, Pierce protease inhibitor cocktail (Pierce A32955, Thermo Fisher Scientific, United States), 0.5 mM DTT]. After desalting by gel filtration using spin columns (Zeba spin desalting columns, 7K MWCO, Thermo Fisher Scientific, United States), the protein concentration was determined by Bradford assay (BioRad protein assay, BioRad, United States). Reactions were performed in triplicate and initial rates were calculated from the absorbance decrease (340 nm) using a molar extinction coefficient of 7.06 mM^–1^ cm^–1^, which comprises both NAD(P)H and GSNO/SNO-CoA absorbance (Sanghani et al., 2006). The linear rate of the reaction was corrected to a reference rate without nitroso compounds.

### Immunoblotting and Nitrosative Stress Treatment

For immunoblot analysis, proteins were extracted from 4- to 6-week-old soil grown WT Col-0 and *hot5-2* plants as described previously (Kim et al., 2012; Liu et al., 2021). Proteins were separated by SDS-PAGE using 12% acrylamide gels and electrophoretically transferred onto nitrocellulose membranes (45 μm, GE Healthcare, United States), blocked with 10% (w/v) fat-free milk, and probed with primary antibodies (anti-AKR4C8/C9/C10/C11, PHYTOAB, United States; 1:5000; and anti-actin, Agrisera AS13 2640, Sweden; 1:3000) over night at 4°C. Following washing with TBST [TBS containing 0.1% (v/v) Tween-20], membranes were incubated with secondary HRP-conjugated antibody (anti-rabbit IgG, PHYTOAB, United States; 1:10 000) for 60 min at RT and signals were obtained by enhanced chemiluminescent detection (ECL).

For nitrosative stress treatment, WT Col-0 seedlings were grown in 0.5× MS media [0.5× MS media, 0.5% (w/v) sucrose, 0.5% (w/v) MES, pH 5.7]. At day 10, media was replaced by fresh media and 2.0 or 5.0 mM of NO donors (Diethylenetriamine NON-Oate, DETA/NO, Cayman Chemical, United States; GSNO) or NO scavenger (Carboxy-PTIO potassium salt, CPTIO, Cayman Chemical, United States) were added, and seedlings treated for 3 h at 40–100 μmol m^–2^ s^–1^ and 22°C. Proteins were extracted and separated on 12% NUSEP nUView gels (NUSEP, United States) and blotted to nitrocellulose membranes. After blocking in 10% (w/v) dry milk in TBST, the membranes were incubated overnight at 4°C with primary antibodies anti-AKR4C8 (PHYTOAB, United States; 1:5000) and anti-actin (Agrisera AS13 2640, Sweden; 1:3000), and washed three times with TBST before incubation with HRP-conjugated secondary antibody (anti-rabbit IgG, PHYTOAB, United States; 1:10 000) for 1 h at RT followed by ECL.

## Results

### Absence of *S*-Nitrosoglutathione Reductase Alters Leaf Protein Composition and Increases Specific Aldo-Keto Reductases

**FIGURE 1:**
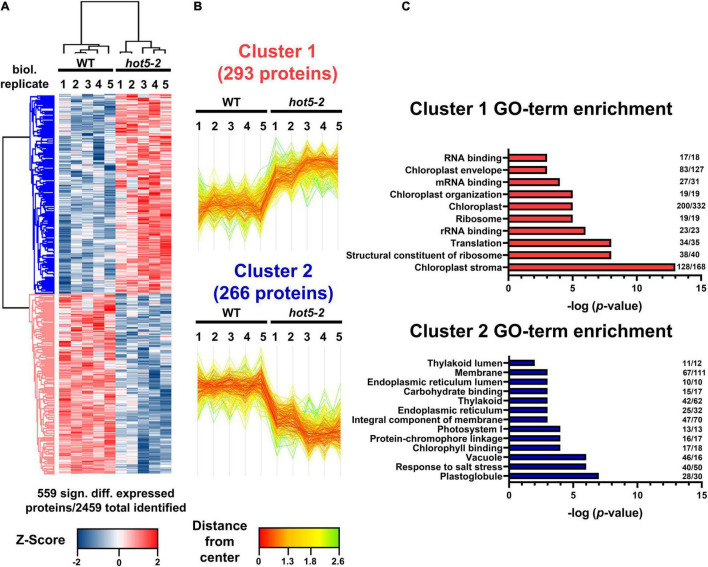
Quantitative proteomic analysis of WT and *hot5-2* leaf material. **(A)** Heatmap of differentially expressed proteins in WT Col 0 and *hot5-2* leaves from 4- to 6-week-old soil grown plants. Five biological replicates were used per genotype. LFQ intensities were *z*-scored prior to Euclidean distance-based hierarchical clustering with Perseus. Five hundred and fifty-nine from a total of 2469 detected proteins are differentially regulated (FDR 5%). Blue and red indicate a lower and higher abundance for each protein in the samples. **(B)** Specific expression patterns in the leaf proteome dataset. Two clusters with 293 and 266 differentially regulated proteins show up- (cluster 1) or downregulation (cluster 2), respectively. **(C)** Gene ontology (GO) enrichment of clusters. *p*-Values of significant GO-terms regarding biological processes, molecular functions, and cellular compartment were –log transformed and displayed in bar graphs. Numbers at the end of each bar represent the number of proteins in that cluster that are associated with the specific GO term. A list of the differentially regulated proteins is provided in Supplementary Data Set 1.

Mutation of the *GSNOR/HOT5* gene (At5g43940) has pleiotropic effects, altering plant growth and development as well as plant reproduction (Lee et al., 2008; Kwon et al., 2012; Xu et al., 2013; Shi et al., 2015). GSNOR is suggested to be the major regulator of NO homeostasis by acting in the specific NADH-dependent degradation of GSNO. Although RNS accumulation and enhanced nitrosative stress (e.g., increased level of low-molecular and protein-SNOs) are observed in *hot5-2* plants, it is not clear how higher GSNO leads to the observed mutant phenotypes. To investigate further how the absence of GSNOR might affect plant phenotypes through altering the expression of other proteins, we examined the total proteome of leaf material from *hot5-2* and WT plants using quantitative proteomics. Total proteins were prepared from five biological replicates of 4- to 6-week-old soil grown WT and *hot5-2* plants (Supplementary Figure 1A) and subjected to shotgun mass spectrometric analysis. A total of around 2500 proteins were identified across all samples (Supplementary Figure 1B and Supplementary Data Set 1), and statistical correlation analysis revealed high Pearson correlation coefficients between replicates (Supplementary Figure 1C). However, principal component analysis showed distinct clustering of WT and *hot5-2* samples, indicating that there are significant differences in protein composition between the genotypes (Supplementary Figure 1D). Approximately 23% of the identified proteins (559 proteins) were differentially regulated in *hot5-2* compared to WT and could be divided into two clusters (Figures 1A,B and Supplementary Data Set 1). Cluster 1 (293 proteins) represents proteins that are significantly upregulated, while cluster 2 (266 proteins) comprises proteins that are downregulated in *hot5-2*. GO-term analysis (Figure 1C) revealed that upregulated proteins in cluster 1 were associated with chloroplasts and chloroplast organization along with RNA binding and translation-related components. Down-regulated proteins in cluster 2 also included chloroplast terms, dominated by chloroplast membrane-related terms.

**FIGURE 2:**
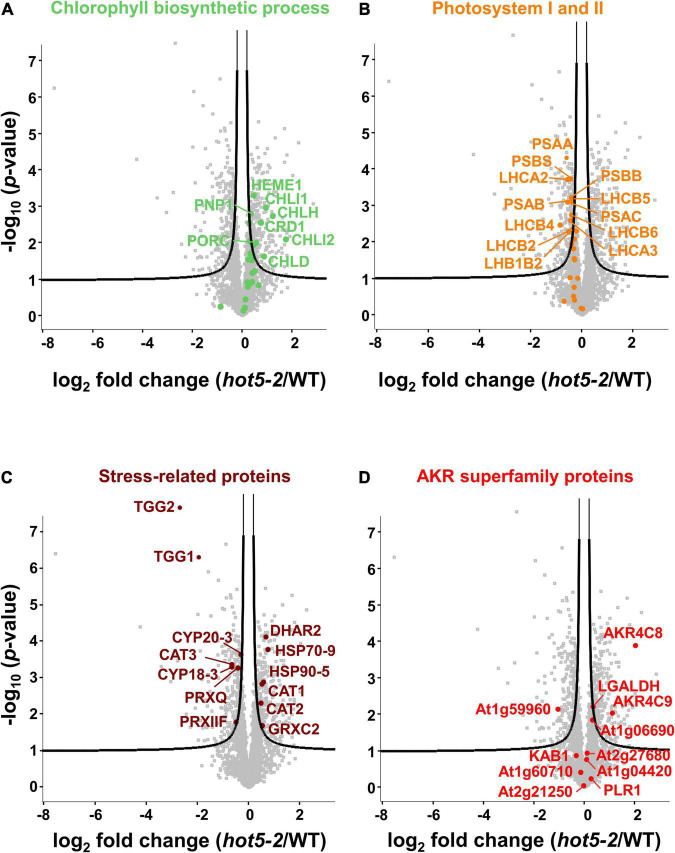
Volcano plots of differentially expressed proteins. Proteins associated with chlorophyll biosynthesis (**A**; GOBP term: chlorophyll biosynthetic process), photosystem I and PSII (**B**; GOCC: photosystem I and PSII), and selected stress-related proteins **(C)** are illustrated. **(D)** Proteins belonging to the AKR protein superfamily (PFAM ID: PF00248) are labeled. The –log 10 of the corrected *p*-values is plotted against the log 2-fold change (FC) in protein levels. FDR cutoff (5%) is indicated by black lines. A list of the significantly regulated proteins is provided in Supplementary Data Set 1.

We further evaluated the proteomics dataset to visualize the most highly regulated proteins using volcano plot analysis (Figure 2 and Supplementary Data Set 1). As reflected in the GO-term search for chloroplast-associated terms, key proteins involved in chlorophyll biosynthesis such as magnesium chelatases CHLI1 and CHLI2 and protochlorophyllide oxidoreductase C (PORC) were upregulated (Figure 2A), while both nuclear and chloroplast-encoded subunits of the photosystem I reaction center PSI (e.g., PSAA, B and C, PSAL, and PSAF) and of photosystem II (e.g., PSBA, B, C, D and S, and LHC proteins) were down regulated (Figure 2B and Supplementary Data Set 1). We also observed differential regulation of stress and redox related proteins in the *hot5-2* mutant (Figure 2C). While some proteins such as catalases (CAT1 and CAT2), dehydroascorbate peroxidases (DHAR2), heat shock proteins (HSP70 and HSP90), and glutaredoxins (GRXC2) are upregulated, other stress responsive proteins belonging to the peroxidase family (peroxiredoxins IIF and Q), cyclophilins (CYP18-3 and CYP20-3), and myrosinases (TGG1 and TGG2) are less abundant in *hot5-2*. Taken together, this suggests that tightly controlled NO homeostasis by GSNOR is important in the regulation of chloroplast processes and the general stress response.

The recent recognition that human AKR1A1 is involved in GSNO metabolism led us to also examine our data for changes in proteins belonging to the AKR superfamily; the disruption of NO metabolism in *hot5-2* plants might impact expression of these enzymes, which are proposed to be involved in NO homeostasis (Stomberski et al., 2019b). Using the PFAM identifier PF00248^1^ (for AKR domain-containing protein), out of the 31 proteins listed for this superfamily in *A. thaliana*, we found 11 proteins in our data set (Figure 2D, Table 1, and Supplementary Data Set 1). Notably, two proteins in the class 4C AKR protein family (AKR4C), AKR4C8 (At2g37760), and AKR4C9 (At2g37770), were significantly upregulated in *hot5-2* (>2- and >1-fold, respectively; Table 1). The only other AKR superfamily member that showed a statistically significant increase (though <0.5-fold) was L-galactose dehydrogenase (LGALDH), which is a well-characterized enzyme of the ascorbate biosynthesis pathway (Smirnoff and Wheeler, 2000).

**TABLE 1:** Aldo-keto reductase superfamily (PFAM ID PF00248) proteins identified in the complete proteomics data set ranked by their log 2 fold change *hot5-2*/WT.

Significant	*p*-Value (−log 10)	Difference (log 2 FC)	Protein names	Gene names/AGI
+	3.84	2.04	Aldo-keto reductase family 4 member C8	AKR4C8/At2g37760
+	2.01	1.12	Aldo-keto reductase family 4 member C9	AKR4C9/At2g37770
+	2.18	0.36	L-Galactose dehydrogenase	LGALDH/At4g33670
	1.83	0.33	Uncharacterized oxidoreductase, chloroplastic	At1g06690
	0.23	0.27	Pyridoxal reductase, chloroplastic	PLR1/At5g53580
	0.92	0.12	/	At2g27680
	0.75	0.10	/	At1g04420
	0.05	−0.02	/	At2g21250
	0.41	−0.14	Probable aldo-keto reductase 4	At1g60710
	0.86	−0.31	Probable voltage-gated potassium channel subunit beta	KAB1/At1g04690
+	2.12	−1.04	/	At1g59960

*Significance as indicated is based on T-test with a permutation-based FDR of 5%.*

### Class 4C AKR Proteins Share Common Features With Human Aldo-Keto Reductase

To understand the relationship of AKR4C8 and 9, as well as other *A. thaliana* AKRs to human AKR1A1, we recovered the closest homologs through a BLAST search of the *A. thaliana* protein database. Notably, AKR4C8 and 9, along with *A. thaliana* homologs AKR4C10 and 11, have the highest amino acid sequence identity to the human protein (Supplementary Table 2). Of the other AKRs identified in the proteomics data set, only At1g59960 and At2g21250 have greater than 25% sequence identity to AKR1A1, but these proteins were either down regulated or unchanged, respectively, in *hot5-2* compared to WT (Figure 2D).

**FIGURE 3:**
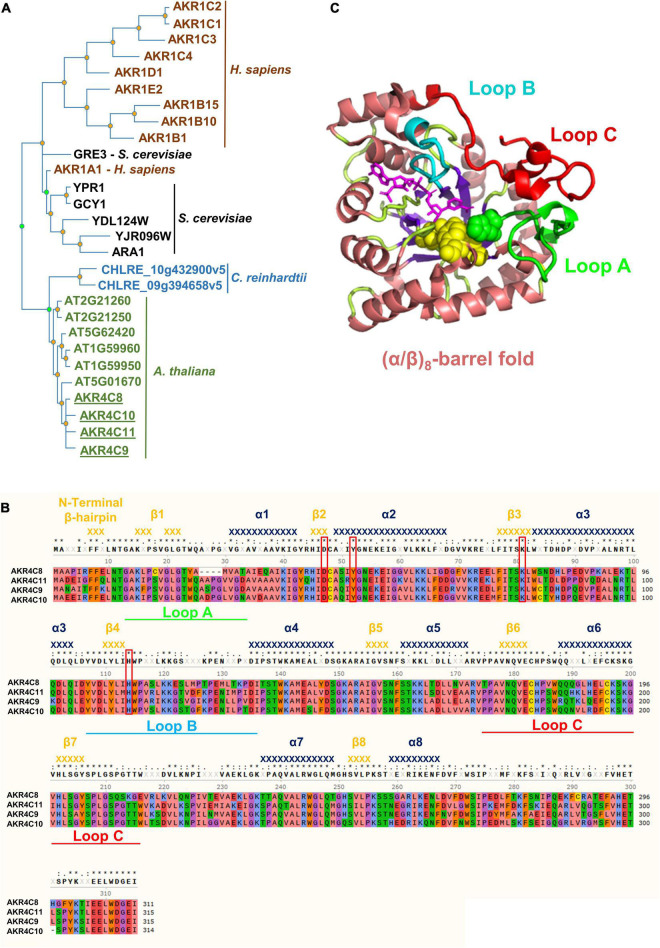
Phylogenetic tree, structure, and amino acid alignment of *A. thaliana* AKR4C proteins. **(A)** Phylogenetic tree of AKRs was constructed by searching for aldo-keto reductases (PTHR11732) in the Panther database using the online tool PhyloGenes. Organisms included were *Chlamydomonas reinhardtii*, *Saccharomyces cerevisiae*, *Homo sapiens*, and *A. thaliana*. UniProt identifier: AKR1C2, P52895; AKR1C1, Q04828; AKR1C3, P42330; AKR1C4, P17516; AKR1D1, P51857; AKR1E2, Q96JD6; AKR1B15, C9JRZ8; AKR1B10, O60218; AKR1B1, P15121; GRE3, P38715; AKR1A1, P14550; YPR1, Q12458; GCY1, P14065; YDL124W, Q07551; YJR096W, P47137; ARA1, P38115; CHLRE_10g432900v5, A0A2K3D9Z5; CHLRE_09g394658v5, A0A2K3DEH4; AT2G21260, Q9SJV1; AT2G21250, Q9SJV2; AT5G62420, Q9FJK0; AT1G59960, Q9SXC0; AT1G59950, Q1PFI5; AT5G01670, F4K9G7; AKR4C8, O80944; AKR4C10, Q84TF0; AKR4C11, Q9M338; and AKR4C9, Q0PGJ6. **(B)** Multiple sequence alignment of AKR4Cs from *A. thaliana*. Red boxes denote the catalytic tetrad residues, while green, cyan, and red bars highlight the flexible loops defining the active site important for substrate specificity. Secondary structure elements [α-helices (dark blue) and β-strands (orange)] were assigned using the structural information of *A. thaliana* AKR4C8 (PDB code 3h7r). Residues are color-coded based on their properties: red, negative; blue, positive; green, hydrophilic; orange, aromatic; purple, conformationally special; salmon, aliphatic/hydrophobic; and yellow, cysteine. **(C)** 3D structure of *A. thaliana* AKR4C8 (PDB code: 3h7r). AKRs share a common (α/β)_8_-barrel structural motif (α-helices in salmon; β-strands in purple) with three flexible loops A (green), B (cyan), and C (red).

We used the AKR proteins in Supplementary Table 2 to construct a phylogeny including other human AKR proteins, along with selected AKR proteins from *Saccharomyces cerevisiae* and *Chlamydomonas reinhardtii* (Figure 3A). The tree clearly shows that the *A. thaliana* AKR4C proteins group together and that the plant and algal proteins have evolved independently from the human and yeast proteins. The phylogeny does not support a closer relationship between human AKR1A1 and any specific *A. thaliana* AKR protein. However, the increase in AKR4C8 and 9 in the *hot5-2* mutant and higher sequence identity/similarity of these proteins to AKR1A1 (Supplementary Table 2) led us to focus further work on the AKR4C proteins.

Notably, the four AKR4C proteins are the most well-described plant AKRs with respect to their structure and *in vitro* oxidoreductase activity on a variety of substrates, though not including GSNO or SNO-CoA (Simpson et al., 2009; Saito et al., 2013). Their high degree of sequence identity is illustrated in Figure 3B, and atomic structures are available for both *A. thaliana* AKR4C8 (Figure 3C) and AKR4C9 (Simpson et al., 2009). They share the native fold that is typical of the AKR superfamily, consisting of a (α/β)_8_-barrel (TIM barrel) with two additional α-helices on the periphery of the enzyme (Jez et al., 1997; Simpson et al., 2009). Three flexible, surface loops (A, B, and C, Figures 3B,C) are involved in defining the specificity of these enzymes toward the substrate, and the flexibility of these loops appears to allow interaction with multiple substrates (Simpson et al., 2009). Another conserved feature are four critical residues, Asp47, Tyr52, Lys81, and His114 (based on residue numbering in *A. thaliana* AKR4C8; Figure 3B), that form the catalytic tetrad (Mindnich and Penning, 2009).

### *Arabidopsis* 4C AKR Proteins Catalyze NADPH-Dependent *S*-Nitrosoglutathione and *S*-Nitroso-Coenzyme a Degradation

**FIGURE 4:**
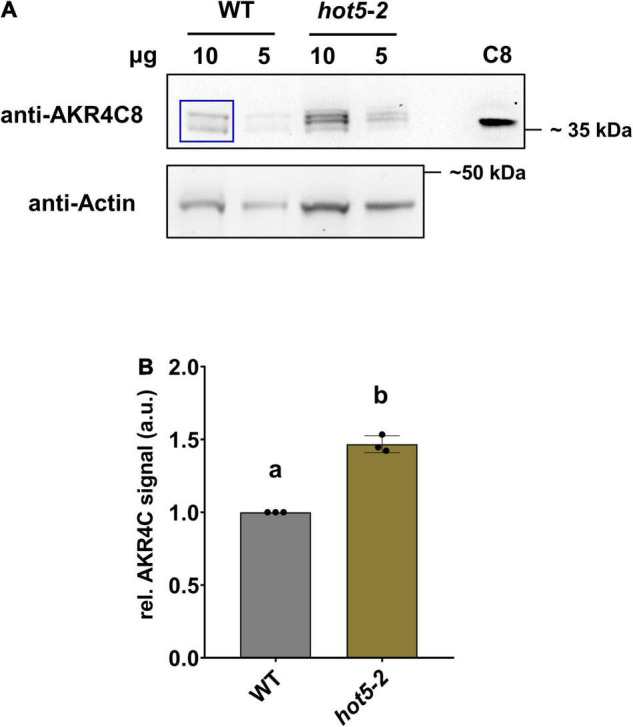
Aldo-keto reductases (AKRs) are increased in *hot5-2*. **(A)** Detection of AKR4C proteins in leaves. Shown is a representative immunoblot from three independent experiments. Total protein extracts (10 and 5 μg) from leaf material were separated by SDS-PAGE. Immunoblots were probed with anti-AKR4C8 polyclonal antibodies (PHYTOAB, 1:5000 dilution) and anti-rabbit IgG HRP-conjugated (PHYTOAB, 1:10 000 dilution). Additionally, 10 ng of purified AKR4C8 protein was loaded (C8). Actin (Agrisera AS13 2640; first AB, 1:3000 dilution; second anti-rabbit-HRP, 1:10 000) was used as loading control. **(B)** Relative quantification of AKR4C. Relative AKR4C signal intensities from the 10 μg samples were quantified using ImageJ (blue inset in **A**). Values were normalized against actin and data represent the mean ± SD calculated from three independent experiments. Different letters indicate groups of *T*-test significant differences of *p* ≤ 0.01.

To enable further studies of their expression levels and enzymatic activities, we cloned and purified all four *A. thaliana* AKR4C proteins (Supplementary Figure 2). We raised antibodies against each of the four proteins individually, tested their specificity and reactivity, and used them to confirm AKR4C levels are increased in *hot5-2* as seen in the proteomics data. Not surprisingly, given the sequence similarity of the four proteins, antibodies against each AKR4C reacted with all four proteins (Supplementary Figure 3). We chose the anti-AKR4C8 antisera to probe for differences in AKR4C levels in *hot5-2* compared to WT. Consistent with the proteomics data, we found an increase in AKR4C signal in the immunoblot analysis, where four different reactive bands were visualized with anti-AKR4C8 antisera, two of which were significantly increased (Figure 4A). We cannot definitively identify the individual bands, but quantification of all four bands indicates a minimum of a 50% increase compared to WT (Figure 4B), supporting the increases seen in the proteomics data.

**FIGURE 5:**
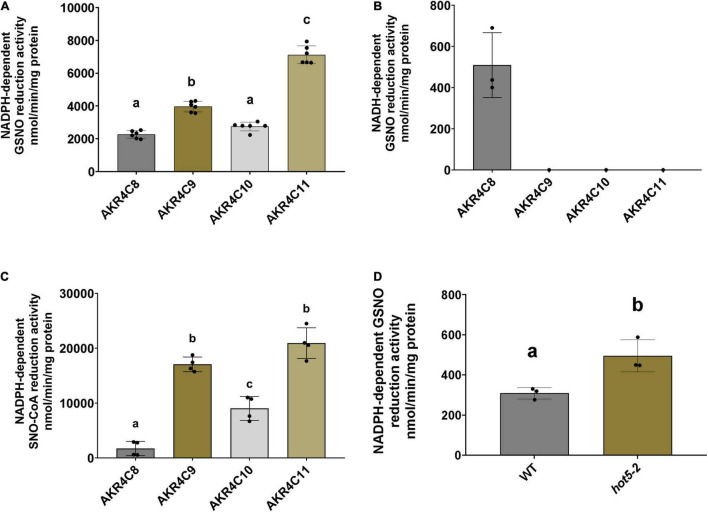
AKR4Cs from *A. thaliana* exhibit GSNO reductase activity and NADPH GSNO reductase activity is increased in *hot5-2* plants. Determination of NADPH- **(A)** and NADH-dependent **(B)** GSNO degradation activity by purified AKR4C proteins, respectively. **(C)** SNO-CoA reduction by AKR4C proteins using NADPH to provide reducing equivalents. Enzyme assays were performed at least in triplicate using two independent protein preparations. The bars represent means ± SD. For panels **(A,C)** different letters indicate groups of significant differences at *p* ≤ 0.05 calculated by one-way ANOVA with *post hoc* Tukey HSD. **(D)** NADPH-dependent *in planta* GSNO reduction activity of leaf extracts from 4- to 6-week-old WT and *hot5-2* plants. Data represent the mean ± SD from three independent experiments. Different letters indicate significant differences of *p* ≤ 0.05 calculated by unpaired *T*-test.

Considering the global conservation of structural and catalytic-related elements of the AKR4C proteins with human AKR1A1 (Supplementary Figure 4), we determined the ability of these proteins to catalyze GSNO/SNO-CoA degradation. The NAD(P)H-dependent activity of purified enzymes was tested in presence of both nitroso-compounds. As shown in Figure 5A, all *A. thaliana* AKR4Cs efficiently degraded GSNO when NADPH was used as a source of reducing equivalents. Specific activities were roughly similar for AKR4C8 and AKR4C10 (2.27 and 2.76 μmol min^–1^ mg^–1^, respectively), while AKR4C9 and AKR4C11 were more efficient with specific activities ∼1.5- and ∼3-fold higher compared to AKR4C8/C10 (3.98 and 7.12 μmol min^–1^ mg^–1^, respectively). The strict specificity toward NADPH dependence was confirmed for all AKR4Cs with the sole exception of AKRC8, which exhibited NADH-dependent GSNO degradation activity that was, however, ∼20% of that measured in the presence of NADPH (0.51 μmol min^–1^ mg^–1^, Figure 5B). To test the substrate specificity of AKR4Cs, we monitored NADPH oxidation in the presence of SNO-CoA, replacing GSNO. As shown in Figure 5C, we observed the lowest specific activity for AKR4C8, whereas the other AKR4Cs were more efficient, with three- to fourfold higher activities than those measured in the presence of GSNO. Altogether, these results demonstrate that AKR4Cs are capable of degrading GSNO, but with opposite specificity toward the cofactor (NADPH vs. NADH) and with 20- to 60-fold lower specific activities than plant GSNORs (Guerra et al., 2016; Tagliani et al., 2021). Moreover, SNO-CoA appears as a suitable substrate for all *A. thaliana* AKR4Cs as observed for human AKR1A1 (Stomberski et al., 2019a).

Given the ability of *A. thaliana* AKR4Cs to catalyze GSNO degradation and the overexpression of AKR4C members in plants lacking GSNOR, we assessed NADPH-dependent GSNO degradation activity of leaf protein extracts from 4- to 6-week-old WT and *hot5-2* plants (Figure 5D). We observed a ∼ 2-fold increase in GSNO degradation in the mutant lacking GSNOR, suggesting that the upregulation of specific AKR4C proteins in *hot5-2* is responsible for the higher NADPH-dependent GSNO reduction. Thus, AKR4C activity may play a significant role in GSNO homeostasis partially compensating for the lack of GSNOR.

## Discussion

Nitrosative stress caused by biotic and abiotic factors is a major challenge to plant survival (Corpas et al., 2021; Sun et al., 2021). The levels of GSNO, a bioactive form of NO, are known to be regulated by GSNOR (Lee et al., 2008; Guerra et al., 2016), and plants lacking the enzyme show multiple phenotypic alterations, indicating that tightly controlled RNS levels are crucial for proper plant growth and development. We compared the total protein profile of a GSNOR null mutant, *hot5-2*, and WT *A. thaliana* leaf material in order to identify proteins that change in expression level as a consequence of accumulation of GSNO and protein-SNOs and might, therefore, be involved in the regulation of these phenotypes. Among the 559 differentially regulated proteins, GO term enrichment analysis of up- and down-regulated clusters revealed that proteins associated with chloroplasts and photosynthesis are differentially regulated in *hot5-2*. We identified components of PSI such as PSAN, PSAF, PSAA, and PSAB as well as components of PSII (PSBD and PSBS) as being significantly downregulated in the null mutant in comparison to WT, in line with the overall reported decrease in non-photochemical quenching in *hot5-2* (Hu et al., 2015). One possible explanation for the altered dissipation of light energy relies on the possibility that chloroplast proteins, including components of the photosystems, are putative targets of S-nitrosation as observed in GSNOR null seedlings (Hu et al., 2015) and *A. thaliana* or *Chlamydomonas* cell suspension cultures treated with GSNO (Lindermayr et al., 2005; Morisse et al., 2014). Consistently, Vanzo et al. (2014) observed an increased S-nitrosation of photosynthesis-related proteins in poplar exposed to ozone stress, suggesting that the photosynthetic process can be under the control of this type of redox PTM in plants.

In contrast, we found an up-regulation of several proteins involved in chlorophyll biosynthesis, such as CHLI1 and CHLI2 as well as PORC. CHLI1 and CHLI2 are chelatases that catalyze the insertion of the magnesium ion into protoporphyrin IX, a key step in chlorophyll metabolism (Ikegami et al., 2007), while PORC is reported to be important for the reduction of protochlorophyllide a to chlorophyllide *a* (Frick et al., 2003). In addition, PORB, another protochlorophyllide oxidoreductase, was reported to be S-nitrosated in *hot5-2* (Hu et al., 2015). Despite the apparent increase in enzymes involved in chlorophyll biosynthesis, *hot5-2* exhibits lower chlorophyll content when grown under long-day conditions (Lee et al., 2008). These contrasting observations could be explained by the inhibitory role of NO on protein function. For example, the S-nitrosation of a peroxiredoxin (PRXIIE) inhibits its peroxidase and peroxynitrite reductase activity in *A. thaliana* (Romero-Puertas et al., 2007). In addition, the activity of glycolytic GAPDH from *A. thaliana* was reversibly inhibited by S-nitrosation at the catalytic Cys149 (Zaffagnini et al., 2013). Therefore, we can speculate that inhibition through S-nitrosation results in a compensatory increase of chlorophyll-related proteins in *hot5-2*, but with no chlorophyll accumulation due to altered assembly of photosystems. Further analysis, also addressing if the reversibility of this PTM by low-molecular weight thiols like GSH or the thioredoxin-system is altered, is needed to clarify the role of S-nitrosation in regulation of proteins involved in chlorophyll biosynthesis.

Plants lacking GSNOR also exhibit increased disease susceptibility due to impaired salicylic acid-dependent immune signaling (Feechan et al., 2005; Kneeshaw et al., 2014). We identified the myrosinases (β-thioglucoside glucohydrolase, TGG) TGG1 and TGG2, proteins associated with the defense response against biotic stresses, as significantly downregulated in *hot5-2*. These enzymes hydrolyze glucosinolates by catalyzing the cleavage of the thioglucosidic bond, releasing toxic products that negatively affect various microbes and herbivores (Liebminger et al., 2012). Overexpression of TGG1 leads to an enhanced defense response in *A. thaliana* (Zhang et al., 2019), while the *tgg1/tgg2* double mutant is defective in both ABA and methyl jasmonate (MeJA)-induced stomatal closure (Islam et al., 2009). These observations indicate that the biotic stress response together with the plant hormonal homeostasis is compromised in GSNOR null plants.

Nitric oxide also dramatically affects redox balance and proteins involved in redox control. Catalases, dehydroascorbate peroxidases, peroxiredoxins, glutaredoxins, and thioredoxins are key components of the antioxidant defense system (Noctor and Foyer, 1998; Mhamdi et al., 2010; Dietz, 2011; Meyer et al., 2012; Müller-Schüssele et al., 2020). We identified differential regulation for some of those proteins. CAT1 and CAT2, for example, are important enzymes in protecting cells from oxidative damage by scavenging H_2_O_2_ (Palma et al., 2020) and show upregulation in our dataset. In contrast, CAT3 is less abundant in *hot5-2*. Interestingly, CAT3 [repressor of *gsnor1* (*rog1*)] has recently been reported to partially suppress the developmental *hot5-2* phenotype, even though the precise molecular mechanism is unknown (Chen et al., 2020). The same study also showed that CAT3 acts as a specific trans-nitrosylase for GSNOR, catalyzing S-nitrosation of Cys10, which in turn leads to the targeted degradation of GSNOR *via* autophagy, suggesting that both enzymes form a positive feedback loop to modulate intracellular NO levels and thereby regulate multiple physiological processes (Zhan et al., 2018; Chen et al., 2020).

Importantly, we identified two AKRs, AKR4C8 and C9, among the most upregulated proteins in *hot5-2* leaves (Figure 2D), along with an overall increase in NADPH-dependent GSNO degradation activity in the mutant (Figure 5D). We speculate that AKRs are upregulated in response to the absence of GSNOR to metabolize GSNO and possibly SNO-CoA. Though there are reports on the activity of purified AKR proteins in reducing diverse substrates, less is known about the importance of these proteins *in planta* (Saito et al., 2013). Overexpression of *Pseudomonas* AKR1 increased salt tolerance in tobacco (Vemanna et al., 2017), and barley plants with elevated *A. thaliana* AKR4C9 exhibited increased salt and cadmium stress tolerance (Éva et al., 2014). Altered stress tolerance was observed in *Physcomitrium* AKR1A knockout mutants, which were more sensitive to NaCl and methylglyoxal treatment (Chen et al., 2019), while the overexpression of an AKR protein from the glyphosate-resistant weed *Echinochloa colona* improved resistance to glyphosate application in transgenic rice (Pan et al., 2019). There have been no studies of specific AKR mutants in *A. thaliana*, and no previous work considering their potential involvement in NO homeostasis. However, AKR4C8 and 9 are highly induced upon diverse stresses, including salinity, drought, and hypoxia, while AKR4C10 and AKR4C11 are less responsive, but are expressed at certain developmental stages (Sengupta et al., 2015; Vemanna et al., 2017; Niranjan et al., 2021). In addition, AKR4C8 and 9 expression, as documented in the Genevestigator database, is highly induced upon multiple biotic and abiotic stresses in comparison to the other AKRs in our proteome experiment, indicating that these two enzymes might be involved in the general stress-response in *A. thaliana* (Supplementary Figure 5A). The stress responsiveness of AKRC8 and C9 and their increased levels in the GSNOR null mutant, suggests that their increase could be mediated by elevated RNS levels. However, we found no evidence for increased AKR4C proteins in WT seedlings treated with a single high dose of NO donors (Supplementary Figure 5B). All members are likely localized to the cytosol, as they lack a N-terminal organelle targeting sequence. Surprisingly, *A. thaliana* AKR4C9 has been reported to be localized to chloroplasts based on transient expression in *Tradescantia reflexa* epidermal cells of GFP-tagged protein, although it is not predicted to have a chloroplast localization sequence (Yamauchi et al., 2011). This result may be an artifact of over-expression in a heterologous system, and we conclude the AKR4C proteins are cytosolic.

Aldo-keto reductases were initially reported as oxidoreductases with a broad substrate specificity, and our results further demonstrate that all members of the class AKR4Cs are capable of reducing the low molecular weight SNOs GSNO and SNO-CoA *in vitro*, using NADPH as a source of reducing equivalents. This is, to our knowledge, the first report on an NADPH-dependent, GSNO-catabolizing system in addition to the NADH-regulated GSNOR in plants. However, each AKR4C was much less efficient than plant GSNOR, but overall, the combined specific activity of all AKR4C is estimated to be ∼9 times lower than that measured for GSNOR. Consistent with our observations, the lower activity of plant AKR4Cs toward GSNO reflects the degradation of GSNO mediated by human AKR1A1, which occurs with a ∼50-fold lower catalytic efficiency compared to human GSNOR (Liu et al., 2001; Stomberski et al., 2019a). Structural variations in human AKR1A1 and plant AKR4Cs are primarily located in the three flexible loops that define substrate binding, which suggests differences in substrate recognition between plant and mammalian AKRs (Supplementary Figure 4). Further work will be necessary to characterize the specific residues that are involved in binding and recognition of GSNO and SNO-CoA, and testing additional plant AKR proteins for similar activity is warranted. Besides GSNOR and the newly identified AKRs, the thioredoxin system has been reported to be involved in the degradation of GSNO and protein-SNOs (Nikitovic and Holmgren, 1996; Kneeshaw et al., 2014), even though it seems unlikely, given that no thioredoxins could be identified as differentially regulated in *hot5-2* (Supplementary Data Set 1). Nonetheless, the effective involvement of the plant thioredoxin system in GSNO homeostasis remains to be explored *in vitro* and *in vivo*. In addition, differences in the AKR4Cs with respect to tissue specific expression, response to cellular and developmental stimuli, as well as their regulation by nitro-oxidative PTMs will provide insight into the role of these enzymes in regulating NO homeostasis in plants.

In summary, the absence of GSNOR leads to the differential expression of proteins involved in various physiological processes including photosynthesis and chlorophyll biosynthesis, as well as enzymes involved in the general stress response, potentially through elevated cellular SNOs levels and S-nitrosation of critical cysteine residues. Additionally, we identified and characterized a new NADPH-dependent GSNO degradation system in plants in form of specific AKRs. Further investigation will be necessary to understand the mechanisms that, besides GSNOR, regulate NO homeostasis, NO-related signaling, and nitrosative stress responses in plants.

## Data Availability Statement

The datasets presented in this study can be found in online repositories. The names of the repository/repositories and accession number(s) can be found below: https://massive.ucsd.edu, MSV000088017.

## Author Contributions

PTre and JR designed and performed the research, analyzed the data, and wrote the manuscript. GG performed the research. EV and MZ supervised the project, analyzed the data, and wrote the manuscript. All authors were involved in the revision of the manuscript and approved the final manuscript.

## Conflict of Interest

The authors declare that the research was conducted in the absence of any commercial or financial relationships that could be construed as a potential conflict of interest.

## Publisher’s Note

All claims expressed in this article are solely those of the authors and do not necessarily represent those of their affiliated organizations, or those of the publisher, the editors and the reviewers. Any product that may be evaluated in this article, or claim that may be made by its manufacturer, is not guaranteed or endorsed by the publisher.

## Footnotes

1
http://pfam.xfam.org/

## Supplementary Material

Supplementary Figure 1 Proteomic sample analysis of WT and *hot5-2*. **(A)** Soil grown (4- to 6-week-old) WT Col-0 and *hot5-2* plants. Shoot material/leaves from five biological replicates per genotype were used in this study. **(B)** Number of identified proteins per sample after filtration in Perseus (see section “Materials and Methods”). **(C)** Global correlation analysis of identified proteins in WT and *hot5-2* samples. Shown is a multi-scatter plot with Pearson correlation coefficient values indicated at the top of each sector; correlation coefficients between replicates of either genotype were 0.98–0.99, while for comparisons between genotypes the coefficients are 0.96–0.97. **(D)** Principal component analysis (PCA) plot of differentially expressed proteins indicates differences between WT and *hot5-2* samples.

Supplementary Figure 2 Purified AKR4C proteins. Coomassie blue staining of purified AKR4C8, AKR4C9, AKR4C10, and AKR4C11 proteins, treated with (+) or without (−) the reducing agent β-mercaptoethanol [2.5% (v/v)]. Three micrograms of each protein were loaded onto 12% SDS-PAGE gels to assess purity of the recombinant proteins. MW markers are shown at the left.

Supplementary Figure 3 Cross reactivity of AKR4C polyclonal rabbit antibodies. Purified AKR4C proteins (25 ng) were separated by 12% SDS-PAGE and subjected to immunoblotting using polyclonal antibodies raised against each protein as mentioned in section “Materials and Methods.” Theoretical molecular masses of non-tagged proteins are 34.69 kDa for AKR4C8, 35.13 kDa for AKR4C9, 34.91 kDa for AKR4C10, and 35.03 kDa for AKR4C11.

Supplementary Figure 4 Comparison of human AKR1A1 and *A. thaliana* AKR4C proteins. Multiple sequence alignment of human AKR1A1 (Uniprot identifier P14550-1) and *A. thaliana* AKR4C (AT2G37760.2, AKR4C8; AT2G37770.2, AKR4C9; AT2G37790.1, AKR4C10; and AT3G53880.1, AKR4C11) proteins. Red boxes denote the catalytic tetrad residues, while green, cyan, and red bars highlight the flexible loops defining the active site important for substrate specificity. Highlighted in yellow are the residues that are conserved among the mammalian and plant AKR proteins.

Supplementary Figure 5 Expression of AKRs. **(A)** Expression profile of *A. thaliana* GSNOR, AKR4C (C8, C9, C10, and C11), and other AKR genes identified in the Panther database for *A. thaliana* (AGIs: AT1G59950, AT1G59960, AT2G21250, AT2G21260, AT5G01670, and AT5G62420). Selected data are obtained from the perturbations AT_AFFY_ATH1-0 database (perturbations) using Genevestigator (https://genevestigator.com/). **(B)** AKR4C protein expression of 10-day old WT Col-0 seedlings treated with NO donors as mentioned in section “Materials and Methods.” Seedlings were treated for 3 h with a single high dose of NO donors (DETA/NO, GSNO) with or without CPTIO and DETA as negative controls. Total proteins (30 μg) were then extracted and subjected to immunoblot analysis using AKR4C8 and actin antibodies as loading control. Purified AKR4C8 protein (5 ng) was used as an additional control. Shown is a representative result from three independent experiments.

Supplementary Table 1 Primers used in this study.

Supplementary Table 2 Percent amino acid sequence identity/similarity of *A. thaliana* AKR proteins with greatest homology to human AKR1A1 as determined by BLAST. AGI numbers in red are those proteins identified in the proteomics dataset. Highlighted in blue are the percent identity/similarity of AKR4Cs in comparison to human AKR1A1. Data were assessed using Emboss Needle (https://www.ebi.ac.uk/Tools/psa/emboss_needle/) and the EBLOSUM62 matrix with default parameters. Values were rounded to the nearest integer.
