# Supplementary figures and images for: Quantitative Proteome Profiling of a S-Nitrosoglutathione Reductase (GSNOR) Null Mutant Reveals a New Class of Enzymes Involved in Nitric Oxide Homeostasis in Plants
Source: Front Plant Sci. 2021 Dec 7;12:787435. doi: 10.3389/fpls.2021.787435 (PMC8695856; doi:10.3389/fpls.2021.787435)

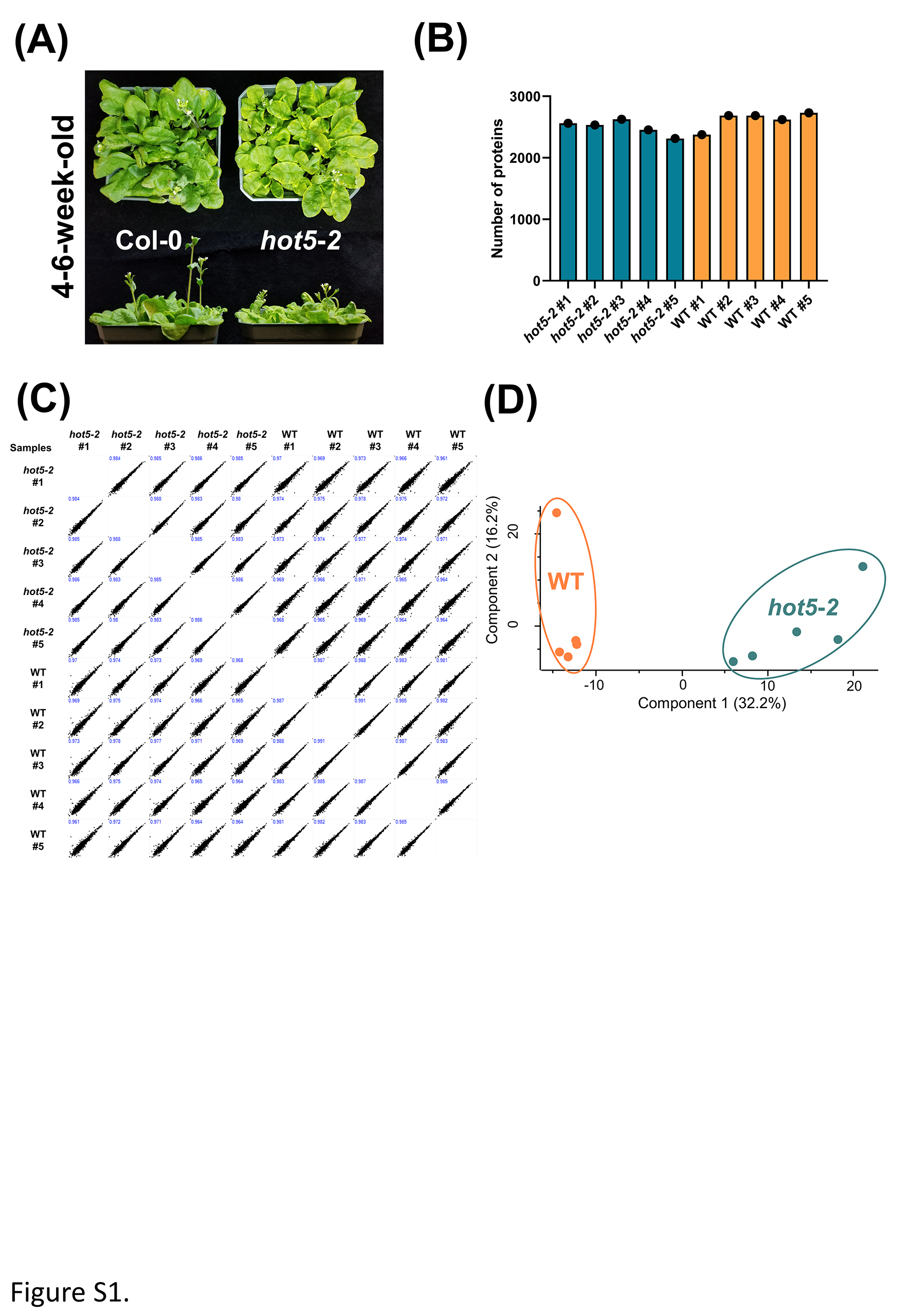

Supplement: Supplementary Figure 1 — Proteomic sample analysis of WT and hot5-2. (A) Soil grown (4- to 6-week-old) WT Col-0 and hot5-2 plants. Shoot material/leaves from five biological replicates per genotype were used in this study. (B) Number of identified proteins per sample after filtration in Perseus (see section “Materials and Methods”). (C) Global correlation analysis of identified proteins in WT and hot5-2 samples. Shown is a multi-scatter plot with Pearson correlation coefficient values indicated at the top of each sector; correlation coefficients between replicates of either genotype were 0.98–0.99, while for comparisons between genotypes the coefficients are 0.96–0.97. (D) Principal component analysis (PCA) plot of differentially expressed proteins indicates differences between WT and hot5-2 samples. [file Image_1.TIF]

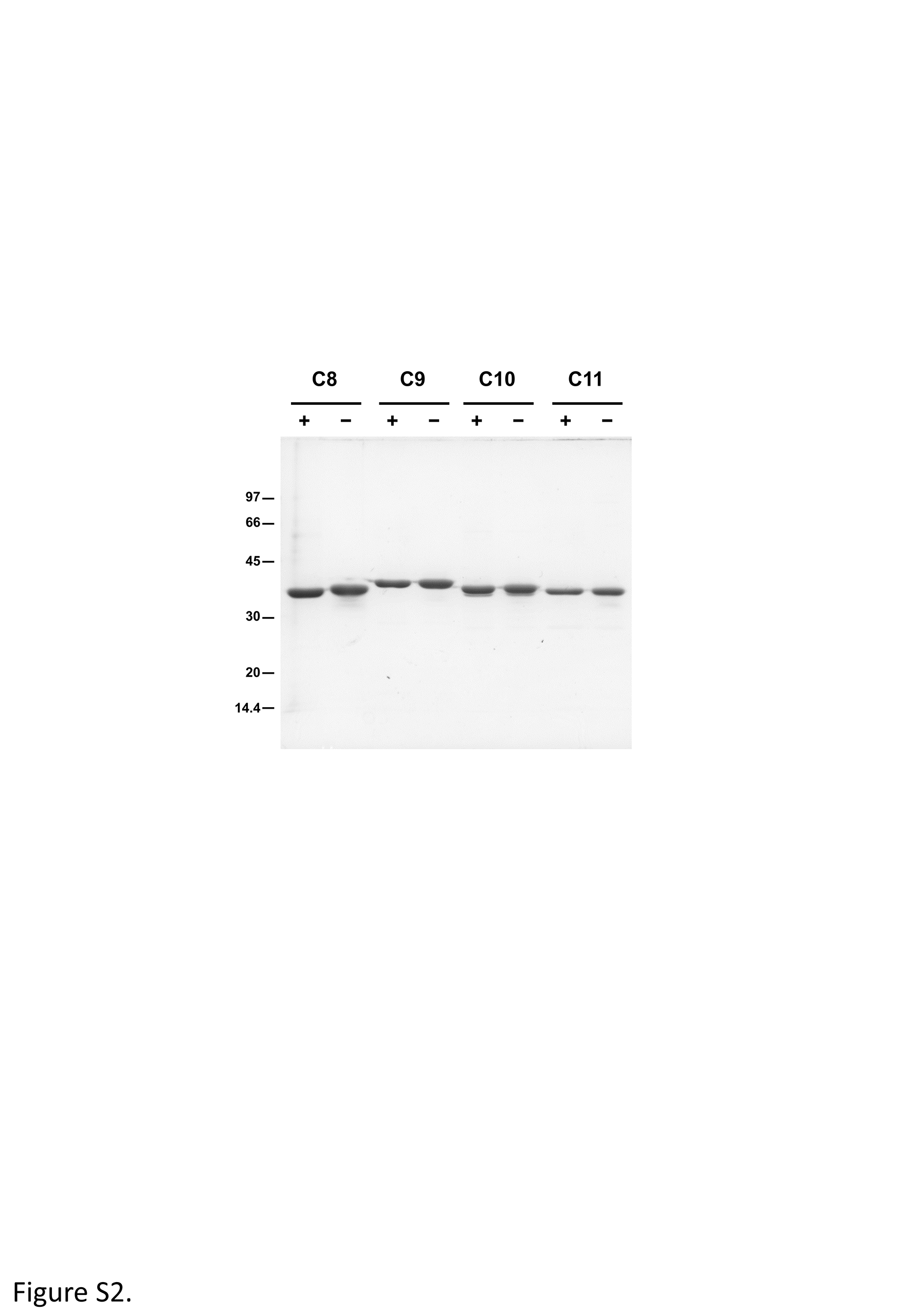

Supplement: Supplementary Figure 2 — Purified AKR4C proteins. Coomassie blue staining of purified AKR4C8, AKR4C9, AKR4C10, and AKR4C11 proteins, treated with (+) or without (−) the reducing agent β-mercaptoethanol [2.5% (v/v)]. Three micrograms of each protein were loaded onto 12% SDS-PAGE gels to assess purity of the recombinant proteins. MW markers are shown at the left. [file Image_2.TIF]

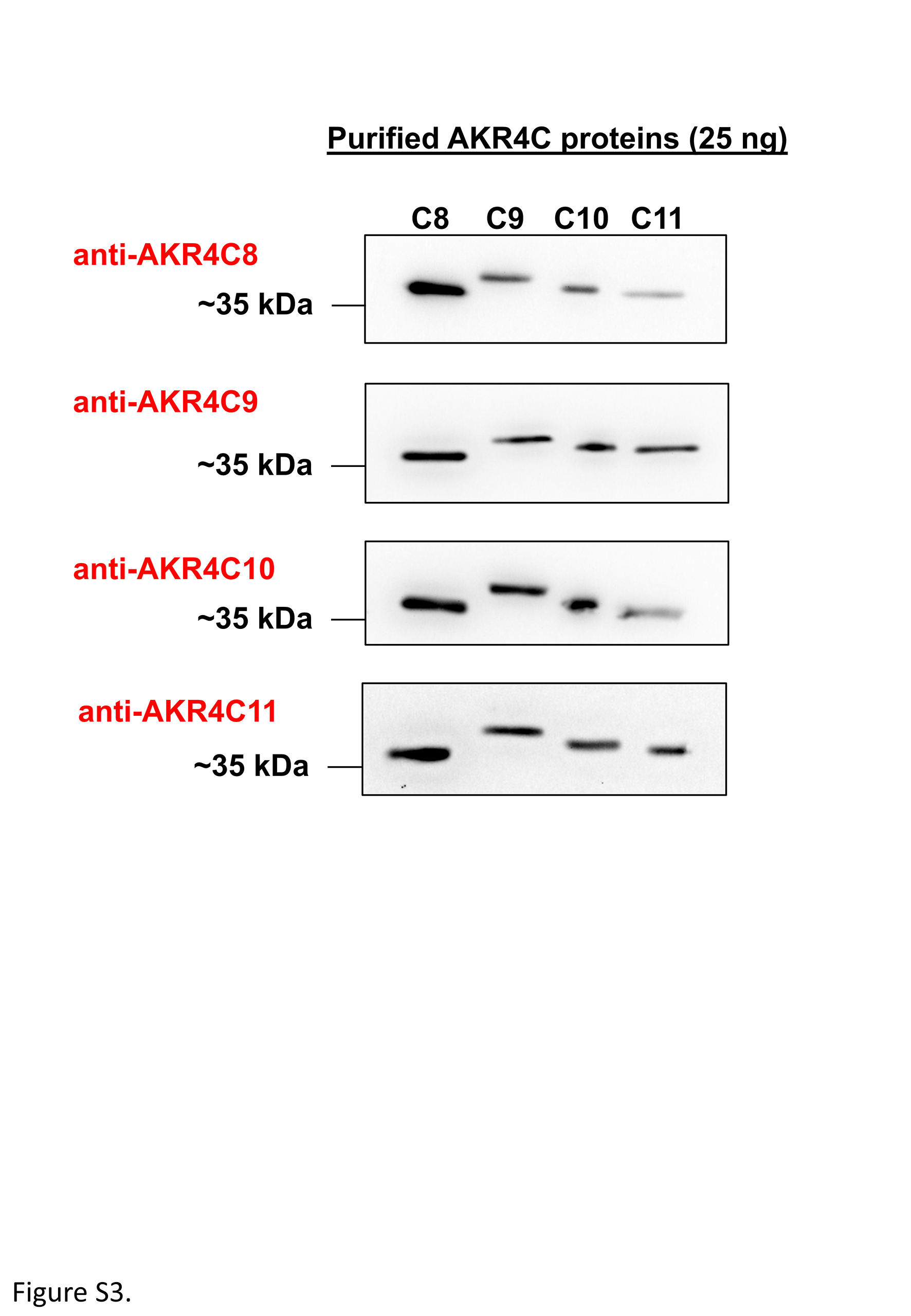

Supplement: Supplementary Figure 3 — Cross reactivity of AKR4C polyclonal rabbit antibodies. Purified AKR4C proteins (25 ng) were separated by 12% SDS-PAGE and subjected to immunoblotting using polyclonal antibodies raised against each protein as mentioned in section “Materials and Methods.” Theoretical molecular masses of non-tagged proteins are 34.69 kDa for AKR4C8, 35.13 kDa for AKR4C9, 34.91 kDa for AKR4C10, and 35.03 kDa for AKR4C11. [file Image_3.TIF]

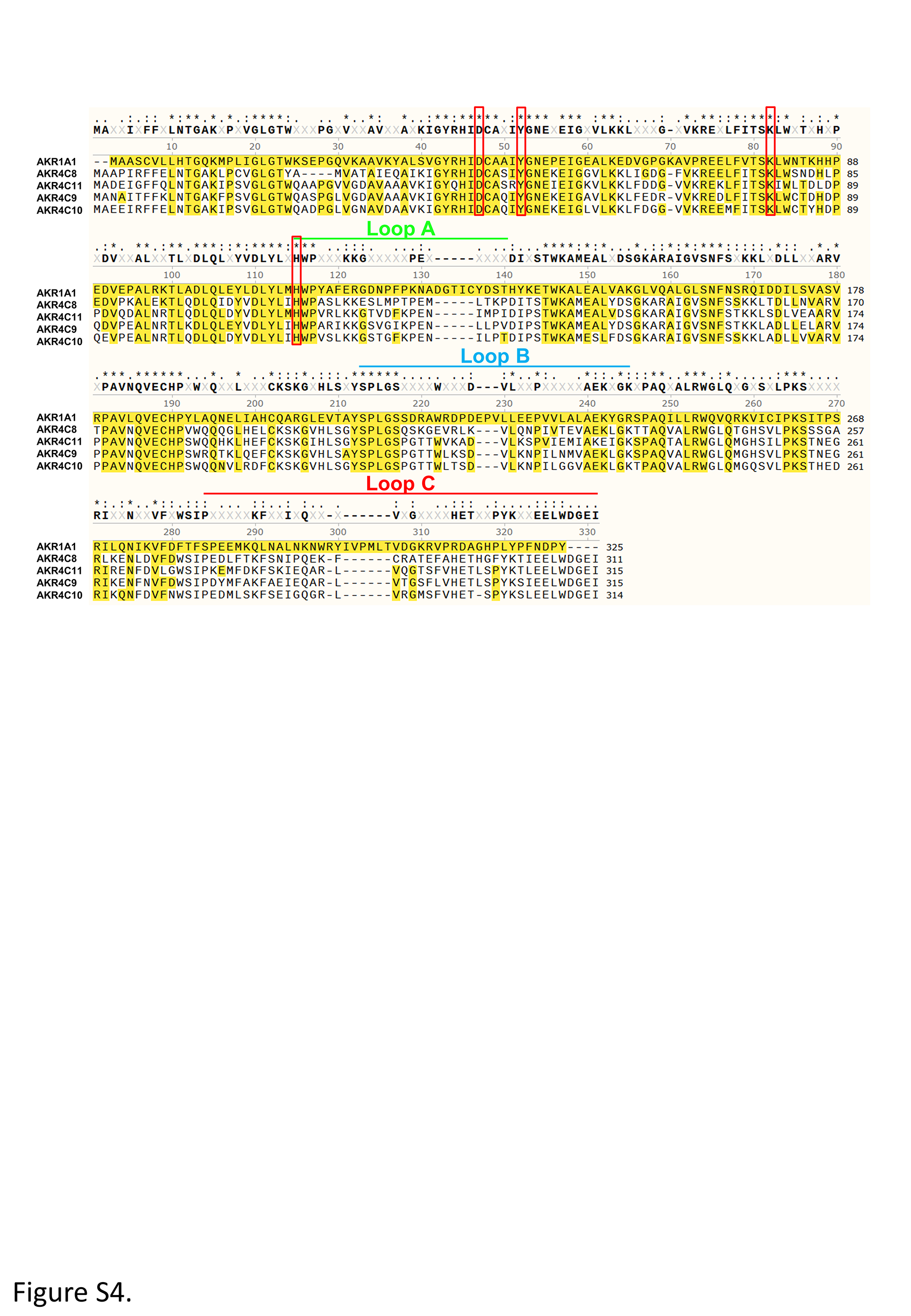

Supplement: Supplementary Figure 4 — Comparison of human AKR1A1 and A. thaliana AKR4C proteins. Multiple sequence alignment of human AKR1A1 (Uniprot identifier P14550-1) and A. thaliana AKR4C (AT2G37760.2, AKR4C8; AT2G37770.2, AKR4C9; AT2G37790.1, AKR4C10; and AT3G53880.1, AKR4C11) proteins. Red boxes denote the catalytic tetrad residues, while green, cyan, and red bars highlight the flexible loops defining the active site important for substrate specificity. Highlighted in yellow are the residues that are conserved among the mammalian and plant AKR proteins. [file Image_4.TIF]

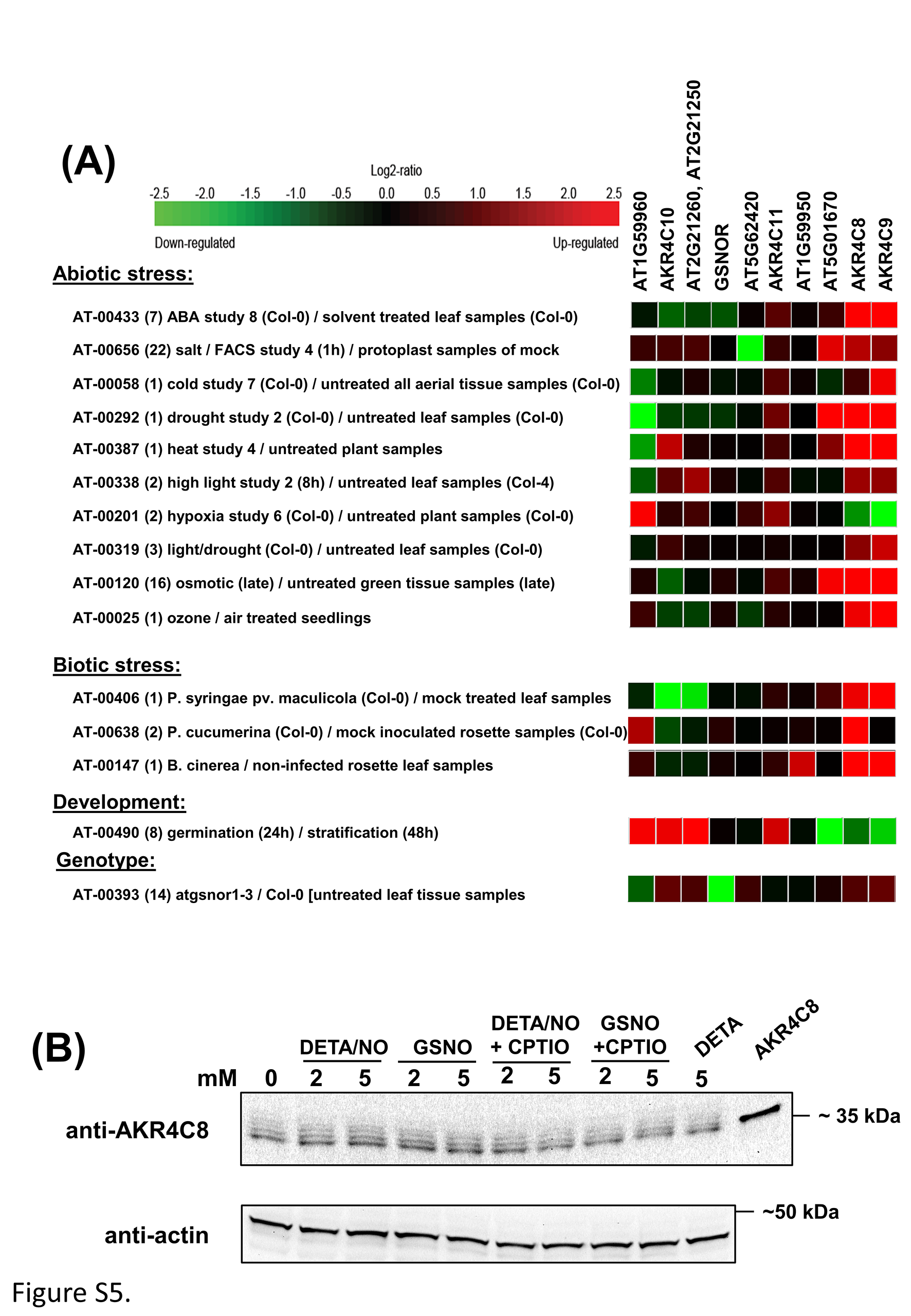

Supplement: Supplementary Figure 5 — Expression of AKRs. (A) Expression profile of A. thaliana GSNOR, AKR4C (C8, C9, C10, and C11), and other AKR genes identified in the Panther database for A. thaliana (AGIs: AT1G59950, AT1G59960, AT2G21250, AT2G21260, AT5G01670, and AT5G62420). Selected data are obtained from the perturbations AT_AFFY_ATH1-0 database (perturbations) using Genevestigator (https://genevestigator.com/). (B) AKR4C protein expression of 10-day old WT Col-0 seedlings treated with NO donors as mentioned in section “Materials and Methods.” Seedlings were treated for 3 h with a single high dose of NO donors (DETA/NO, GSNO) with or without CPTIO and DETA as negative controls. Total proteins (30 μg) were then extracted and subjected to immunoblot analysis using AKR4C8 and actin antibodies as loading control. Purified AKR4C8 protein (5 ng) was used as an additional control. Shown is a representative result from three independent experiments. [file Image_5.TIF]
