# Supplementary material for: Quantitative Proteome Profiling of a S-Nitrosoglutathione Reductase (GSNOR) Null Mutant Reveals a New Class of Enzymes Involved in Nitric Oxide Homeostasis in Plants
Source: Front Plant Sci. 2021 Dec 7;12:787435. doi: 10.3389/fpls.2021.787435 (PMC8695856; doi:10.3389/fpls.2021.787435)
Supplement: Supplementary Table 1 — Primers used in this study. [file Table_1.pdf]

Table S 1. Primers used in this study

Construct/ Gene AGI/ Gene model			Primer Sequence 5′->3′
pET23b-HIS-SUMO backbone	/	Forward	ACCACCGGTCTGTTCTCTGTG
		Reverse	CGAGCACCACCACCACCACCACTG
AKR4C8	AT2G37760.2	Forward	CACAGAGAACAGACCGGTGGTATGGCAGCTCCGATTCGATTCTTTG
		Reverse	CAGTGGTGGTGGTGGTGGTGCTCGTCAAATTTCACCGTCCCACAATTCC
AKR4C9	AT2G37770.2	Forward	CACAGAGAACAGACCGGTGGTATGGCAAATGCGATCACATTTTTCAAG
		Reverse	CAGTGGTGGTGGTGGTGGTGCTCGTCATATCTCGCCATCCCATAATTC
AKR4C10	AT2G37790.1	Forward	CACAGAGAACAGACCGGTGGTATGGCAGAGGAAATTAGATTCTTTG
		Reverse	CAGTGGTGGTGGTGGTGGTGCTCGTCATATCTCACCGTCCCAGAG
AKR4C11	AT3G53880.1	Forward	CACAGAGAACAGACCGGTGGTATGGCGGACGAAATCGGATTC
		Reverse	CAGTGGTGGTGGTGGTGGTGCTCGTCAGATCTCACCATCCCAAAGC
