# Supplementary material for: Quantitative Proteome Profiling of a S-Nitrosoglutathione Reductase (GSNOR) Null Mutant Reveals a New Class of Enzymes Involved in Nitric Oxide Homeostasis in Plants
Source: Front Plant Sci. 2021 Dec 7;12:787435. doi: 10.3389/fpls.2021.787435 (PMC8695856; doi:10.3389/fpls.2021.787435)
Supplement: Supplementary Table 2 — Percent amino acid sequence identity/similarity of A. thaliana AKR proteins with greatest homology to human AKR1A1 as determined by BLAST. AGI numbers in red are those proteins identified in the proteomics dataset. Highlighted in blue are the percent identity/similarity of AKR4Cs in comparison to human AKR1A1. Data were assessed using Emboss Needle (https://www.ebi.ac.uk/Tools/psa/emboss_needle/) and the EBLOSUM62 matrix with default parameters. Values were rounded to the nearest integer. [file Table_2.pdf]

Table S 2. Percent amino acid sequence identity/similarity of *A. thaliana* AKR proteins with greatest homology to human AKR1A1 as determined by BLAST. AGI numbers in red are those proteins identified in the proteomics dataset. Highlighted in blue are the percent identity/similarity of AKR4Cs in comparison to human AKR1A1. Data were assessed using Emboss Needle (https://www.ebi.ac.uk/Tools/psa/emboss_needle/) and the EBLOSUM62 matrix with default parameters. Values were rounded to the nearest integer.

	**Protein**		**AKR1A1**	**AKR4C8**	**AKR4C9**	**AKR4C10**	**AKR4C11**						
		**Uniprot ID / AGI**	**P14550**	**At2g37760**	**At2g37770**	At2g37790	At3g53880	At5g62420	At5g01670	**At1g59960**	**At2g21250**	At2g21260	At1g59950
**1**	**AKR1A1**	**P14550**											
**2**	**AKR4C8**	**At2g37760**	**40/56**										
**3**	**AKR4C9**	**At2g37770**	**40/56**	67/77									
**4**	**AKR4C10**	At2g37790	**43/59**	69/78	81/90								
**5**	**AKR4C11**	At3g53880	**40/58**	64/76	78/90	76/89							
**6**		At5g62420	32/51	42/60	45/63	44/63	42/61						
**7**		At5g01670	33/49	36/52	42/58	38/57	37/56	35/52					
**8**		**At1g59960**	31/51	37/56	38/57	40/59	37/57	39/60	23/33				
**9**		**At2g21250**	37/59	36/53	38/55	37/56	36/57	35/57	24/36	33/56			
**10**		At2g21260	37/59	36/54	36/56	36/56	35/55	34/56	32/50	34/56	92/97		
**11**		At1g59950	34/52	38/58	42/61	42/62	37/57	40/60	33/52	83/90	34/56	34/56	
